# Phenotypic heterogeneity by site of infection in surgical sepsis: a prospective longitudinal study

**DOI:** 10.1186/s13054-020-02917-3

**Published:** 2020-05-07

**Authors:** Julie A. Stortz, Michael C. Cox, Russell B. Hawkins, Gabriela L. Ghita, Babette A. Brumback, Alicia M. Mohr, Lyle L. Moldawer, Philip A. Efron, Scott C. Brakenridge, Frederick A. Moore

**Affiliations:** 1 Department of Surgery, University of Florida College of Medicine, 1600 SW Archer Road, PO Box 100108, Gainesville, Florida 32610-0108 USA; 2 Department of Biostatistics, University of Florida, Gainesville, FL USA

**Keywords:** Surgical sepsis, Site of infection, Phenotypes, Chronic critical illness, Heterogeneity, Long-term outcomes, Functional outcomes, Proinflammation, Immunosuppression, Organ dysfunction

## Abstract

**Background:**

The role of site of infection in sepsis has been poorly characterized. Additionally, sepsis epidemiology has evolved. Early mortality has decreased, but many survivors now progress into chronic critical illness (CCI). This study sought to determine if there were significant differences in the host response and current epidemiology of surgical sepsis categorized by site of infection.

**Study design:**

This is a longitudinal study of surgical sepsis patients characterized by baseline predisposition, insult characteristics, serial biomarkers, hospital outcomes, and long-term outcomes. Patients were categorized into five anatomic sites of infection.

**Results:**

The 316 study patients were predominantly Caucasian; half were male, with a mean age of 62 years, high comorbidity burden, and low 30-day mortality (10%). The primary sites were abdominal (44%), pulmonary (19%), skin/soft tissue (S/ST, 17%), genitourinary (GU, 12%), and vascular (7%). Most abdominal infections were present on admission and required source control. Comparatively, they had more prolonged proinflammation, immunosuppression, and persistent organ dysfunction. Their long-term outcome was poor with 37% CCI (defined as > 14 in ICU with organ dysfunction), 49% poor discharge dispositions, and 30% 1-year mortality. Most pulmonary infections were hospital-acquired pneumonia. They had similar protracted proinflammation and organ dysfunction, but immunosuppression normalized. Long-term outcomes are similarly poor (54% CCI, 47% poor disposition, 32% 1-year mortality). S/ST and GU infections occurred in younger patients with fewer comorbidities, less perturbed immune responses, and faster resolution of organ dysfunction. Comparatively, S/ST had better long-term outcomes (23% CCI, 39% poor disposition, 13% 1-year mortality) and GU had the best (10% CCI, 20% poor disposition, 10% 1-year mortality). Vascular sepsis patients were older males, with more comorbidities. Proinflammation was blunted with baseline immunosuppression and organ dysfunction that persisted. They had the worst long-term outcomes (38% CCI, 67% poor disposition, 57% 1-year mortality).

**Conclusion:**

There are notable differences in baseline predisposition, host responses, and clinical outcomes by site of infection in surgical sepsis. While previous studies have focused on differences in hospital mortality, this study provides unique insights into the host response and long-term outcomes associated with different sites of infection.

## Introduction

Despite decades of intensive research, sepsis remains a common deadly, costly, and debilitating intensive care unit (ICU) syndrome [1–3]. Traditionally, sepsis has been viewed to be due to an over-exuberant systemic inflammatory response syndrome (SIRS) that caused early deaths from refractory septic shock or unremitting multiple organ failure (MOF) [4–6]. Once triggered, this systemic process was believed to progress independent of the inciting infection and thus patients with different types of infection have been grouped together in clinical trials [7]. Unfortunately, innumerable trials of promising immune-modulating interventions have failed to reduce early mortality and the recent consensus is that heterogeneity played a major role in these dismal failures [8–10]. It has been shown that site of infection is an independent predictor of early mortality [11–15]. However, the epidemiology of sepsis has evolved. With rapid implementation of evidence-based care, early mortality has decreased substantially, but many sepsis survivors are now progressing into chronic critical illness (CCI) with poorly defined long-term outcomes [16–22]. Moreover, the differential impact of site of infection on the immune response, progression of organ dysfunctions, and ICU utilization has not been characterized. We, therefore, sought to determine if there were significant differences in the host response and current epidemiology of surgical sepsis categorized by site of infection. We hypothesized that patients classified by site of infection will experience different clinical phenotypes when compared by baseline predisposition, initial septic insult characteristics, serial immune biomarker response, and organ dysfunction resolution, as well as their ICU and long-term clinical outcomes. Understanding this heterogeneity will assist clinicians in prognostication and decision-making. Additionally, insights into the underlying pathobiology of this phenotypic heterogeneity will be needed to develop more precise interventions and design future clinical trials.

## Methods

### Study population rationale

In this study, we define surgical sepsis as being sepsis treated in the surgical and trauma ICUs. Compared to medical ICU sepsis, surgical ICU sepsis patients are less likely to have severe comorbidities because of surgeon selection bias and trauma ICU patients tend to be younger with a less severe comorbid disease. Surgical ICU sepsis patients are also different because they are exposed to sequential insults that promote persistent dysregulated immunity. Our interest in this population originates from two reports in which we demonstrated that ICU mortality after surgical sepsis decreased substantially as a result of early sepsis screening and reliable implementation of evidence-based ICU care [16, 23]. We noted, however, that a substantial portion of the patients were being discharged to non-home destinations with significant disabilities. In a 2012 review article, we described the emergence of this new predominant clinical trajectory of CCI with lingering low-grade organ dysfunction and we coined the term *Persistent Inflammation, Immunosuppression, and Catabolism Syndrome* (PICS) to provide a mechanistic hypothesis in which to study CCI in septic surgical ICU patients [24]. In 2014, the University of Florida (UF) Sepsis Critical Illness Research Center (SCIRC) was awarded a National Institute of General Medical Sciences (NIGMS) P50 team science grant to test this hypothesis. The clinical component of this translational research program was a 5-year prospective longitudinal cohort study which collected the data included in this manuscript. Other reports from this team science effort indicate that PICS-CCI in surgical ICU sepsis and trauma patients is a valid concept [25–27].

### Study design, setting, and participants

This is an analysis of a prospective, longitudinal cohort study that enrolled patients over 4 years ending December 2018 who were then followed for 1 year. The purpose of the study was to define the epidemiology, dysregulated immunity, and outcomes of surgical patients that were admitted with, or subsequently developed sepsis. It was carried out in two 24 bed surgical ICUs at the University of Florida (UF), Shands Hospital (Gainesville, FL; USA), and conducted by the UF SCIRC. A detailed description of the study design with specific inclusion and exclusion criteria as well as the clinical and laboratory standard operating procedure (SOPs) utilized has been published [28]. In brief, overall cohort inclusion criteria included: (1) age ≥ 18 years, (2) clinical diagnosis of sepsis as defined by 2001 international consensus guidelines, and (3) entrance into an electronic medical record sepsis screening and evidence-based ICU management protocol [16, 23]. Exclusion criteria eliminated patients whose baseline immunosuppression, end-stage comorbidities, or severe functional disabilities would be a primary determinant of their long-term outcomes and thus confound outcome assessment. Sepsis screening was performed using the Modified Early Warning Signs-Sepsis Recognition Score (MEWS-SRS), which quantifies derangement in temperature, heart rate, respiratory rate, blood pressure, mental status, and white blood cell count [16]. Patients with significant derangements identified in these variables to exceed screening score of 5 were assessed by a physician or advanced practice provider to confirm the likelihood of sepsis.

Patients believed to be septic underwent appropriate early goal-directed fluid resuscitation as well as the selection and administration of empiric antibiotics based on the presumed site of infection. The patients were managed by a multidisciplinary ICU team that ensured compliance with the electronic medical record-based protocols. The patient study records were adjudicated by the clinical faculty members at weekly sepsis adjudication meetings to ensure the appropriate diagnosis of site of infection and the severity of sepsis. Clinical data were collected into an established database designed to characterize the epidemiology of surgical sepsis including baseline demographics, comorbidities, body mass index (BMI), hospital admission diagnosis, site of infection, sepsis diagnosis, sepsis severity (i.e., sepsis, severe sepsis, and septic shock), need for mechanical ventilation as well as mechanical ventilation, and ICU and hospital days [29]. Infections were defined using CDC definitions and sepsis was classified as “present on admission” if diagnosed within 48 h and “hospital-acquired” if diagnosed after 48 h after admission.

Secondary infections were defined as any probable or microbiologically confirmed bacterial, yeast, fungal, or viral infection requiring treatment with antimicrobials and occurring at least 48 h after sepsis protocol onset during the index hospitalization. Infections within 48 h of sepsis onset were considered coexisting infections and therefore excluded.

The initial predictive mortality of sepsis was assessed by the Acute Physiology Age Chronic Health Evaluation (APACHE) II and Sequential Organ Failure Assessment (SOFA) scores at 24 h. Organ dysfunction progression/resolution was assessed by serial SOFA scores. MOF defined by the Denver MOF score and acute kidney injury (AKI) was defined by Kidney Disease Improving Global Outcomes (KDIGO) score. Patients were classified by three inpatient clinical trajectories: (1) early death, (2) rapid recovery, and (3) chronic critical illness (CCI). Early death was defined as death within 14 days of sepsis onset. CCI was defined as an ICU stay greater than or equal to 14 days with evidence of persistent organ dysfunction based upon components of the SOFA score. Rapid recovery patients are those discharged from the ICU within 14 days with resolution of organ dysfunction, or those not meeting criteria for early death or CCI. Discharge disposition was classified based on known associations with long-term outcomes as either “good” (home with or without health care services, or rehabilitation facility) or “poor” (long-term acute care centers, skilled nursing facilities, another acute care hospital, hospice or inpatient death). Following discharge, patients (or patient proxy) were contacted monthly by telephone to obtain information related to subsequent hospitalizations, and current disposition, including mortality (with cross-check validation via the US Social Security Death Index). Performance status (i.e., physical function) was assessed using the 6-point WHO/Zubrod scale ranging from 0 to 5, with 0 denoting fully active and able to carry on normal physical activity without restriction, 1 for symptomatic subjects but completely ambulatory and able to carry out normal daily activities (light work), 2 for mild disability subjects who were in bed ≤ 50% of daytime and capable of self-care but no work activities, 3 for moderate disability subjects who were in bed for more than half of daytime and capable of limited self-care, 4 for severe disability subjects who were completely bedridden subjects, and 5 denoting death [30].

### Subgroup classification by site of infection

For this report, patients were divided into five groups based on the anatomic site of their inciting infection including (a) abdominal, (b) genitourinary (GU), (c) pulmonary, (d) skin/soft tissue (S/ST), and (e) vascular. Abdominal sepsis included primary infections arising within the abdomen (from the gastrointestinal tract, pancreas, or hepatobiliary tree) or secondary infections from procedural complications (such as anastomotic leaks, iatrogenic perforations, and abscesses). Pulmonary infections include pneumonia and empyema. S/ST infections included primary soft tissue infections and surgical site infections. The diagnosis of necrotizing soft tissue infection (NSTI) was confirmed by examining the operative reports for evidence of necrotic tissue requiring debridement and/or amputation. GU infections included infections arising from the urinary tract or the male or female genital tracts. Vascular infections included mycotic aneurysms, septic thrombophlebitis, infected prosthetic grafts, or a central line-associated bloodstream infections. The latter was considered as any bloodstream infection in a patient with a central line/catheter at the time of, or within 48 h prior to, the onset of infection which does not appear to be related to infection from another site.

### Sepsis staging of subgroups based on PIRO classification [31]

*Predisposition* is characterized by baseline demographics, comorbidities, and reason for admission. *Insult* variables included sepsis present on admission versus hospital-acquired, initial sepsis severity, culture results, and source control interventions. *Response* variables included serial proinflammatory biomarkers, including interleukin (IL)-6 and IL-8, and immunosuppression including soluble program death ligand-1 (sPDL-1) and absolute lymphocyte counts (ALC) obtained on days 1, 4, 7, and 14 after sepsis diagnosis [26]. *Organ dysfunction* variables included MOF, AKI, and serial SOFA scores.

### Outcomes of subgroups

*ICU outcome* variables included ICU days, need for mechanical ventilation, ventilator-free days (determined by subtracting the number of days on mechanical ventilation after sepsis protocol onset from 30), development of secondary infections (presented as mean per patient and adjusted for the time at risk [i.e., secondary infections per 100 hospital person days]), clinical trajectory (early death/rapid recovery/CCI), and discharge disposition. *Long-term outcomes* included 1-year survival probability and performance status by WHO/Zubrod score.

### Healthy control subjects

Age-, gender-, and race/ethnicity-matched healthy control subjects from the surrounding communities (who responded to e-mail requests) were consented, and a single blood sample collected. Limited clinical data were collected on these subjects, but any individual with known history of autoimmune disease, taking immunosuppressive medication, active cancer treatment, or active infection was excluded. We recruited 60 people in order to obtain sufficient sample size for age, gender, and race/ethnicity matching.

### Blood draws and laboratory analyses

Blood samples were collected from septic patients at 24 h and 4, 7, and 14 days after sepsis protocol onset for subjects remaining inpatient and analyzed for biomarkers of inflammation (IL-6, IL-8) and immunosuppression (ALC and sPDL-1). Age- and gender-matched controls had blood samples collected once. Plasma biomarkers concentrations were determined by multiplex or ELISA. Complete blood counts with differential were performed by the Clinical and Diagnostic Laboratories at the University of Florida Health Shands Hospital to determine ALC.

### Statistical analysis

Data are presented as frequency and percentage, mean and standard deviation or standard error, or median and interquartile range. Fisher’s exact test and the Kruskal-Wallis test were used for comparison of categorical and continuous variables, respectively. Measured biomarkers were compared using nonparametric rank tests to determine significant differences between groups at each time point, while within-group differences between time points were analyzed using paired *t* tests. SOFA scores between groups were compared using the Kruskal-Wallis test at a priori selected time points of 1, 4, 7, and 14 days after sepsis onset. SOFA score was imputed for living patients discharged prior to day 14. For patients with a poor discharge disposition, the last available in-hospital component scores were carried forward. Similarly, for patients with a good disposition, the last available in-hospital component scores were used for hepatic, coagulation, and renal component scores, while respiratory and CNS components were assumed to be 0. The log-rank test was used to compare Kaplan-Meier product-limit estimates of survival between groups. All significance tests were two-sided, with a *p* value of ≤ 0.05 considered statistically significant. Statistical analyses were performed using SAS (v.9.4, SAS Institute, Cary, NC). Multivariate logistic regression models were utilized to assess relative risk of 12-month mortality among each site of infection in comparison to pulmonary, while controlling for age, gender, BMI, inter-facility transfer status, Charlson comorbidity index, septic shock, and 24-h SOFA score.

## Results

The study population consisted of 316 septic study patients and 37 healthy age-, gender-, and race/ethnicity-matched control subjects. Table 1 lists five major anatomic sites of infection and specific causes of infection, and Table 2 depicts the baseline predisposition characteristics of the overall cohort and the five subgroups. Overall, the study patients were predominantly Caucasian, roughly half were males, and the mean age was 62 years. Over 40% had three or more comorbidities, the most frequent being hypertension, diabetes, and coronary artery disease. The most common reasons for hospital admission included active infection (60%), followed by elective surgery (20%), trauma (10%), and other chronic medical problems (10%). The five subgroups (in decreasing frequency) were abdominal (44%), pulmonary (19%), S/ST (18%), GU (12%), and vascular (7%). There were subgroup differences in baseline predisposition. Abdominal sepsis patients had the highest rates of comorbid active cancer, defined as solid or hematologic malignancy diagnosed or treated within 6 months (excluding non-melanoma skin cancer) or recurrent/metastatic disease. Pulmonary infections (principally pneumonia) most frequently occurred in males admitted for trauma or elective surgery. S/ST infection patients (predominantly NSTIs) had the highest median BMIs, with a trend toward less comorbid disease although over half of the subjects were diabetic. Vascular infections (predominantly prosthetic grafts infections and mycotic aneurysms) occurred in older male patients with several comorbidities (i.e., coronary artery disease, chronic lung disease, and peripheral vascular disease), and almost all were inter-facility hospital transfers.

**Table 1:** Five major anatomic sites of infection and specific causes

**Abdominal,*n*(%)**	**140 (44%)**
Perforation/peritonitis/bscess	18
Cholecystitis/cholangitis	9
Pancreatitis	9
Small bowel obstruction/ischemia/perforation	13
Appendicitis/diverticulitis/colitis	18
Ischemic bowel	22
Anastomotic leak	16
Intragenic perforation	17
Postoperative abscess/biloma	18
**Pulmonary,*n*(%)**	**59 (19%)**
Pneumonia	55
Empyema	4
**Skin/soft tissue (S/ST),*n*(%)**	**56 (18%)**
Surgical site infection	13
Necrotizing soft tissue infection	37
Non-necrotizing soft tissue infection	6
**Genitourinary (GU),*n*(%)**	**40 (12%)**
Ureter obstruction/instrumentation	22
Urinary tract infection/pyelonephritis	14
Uterine/adnexal	4
**Vascular,*n*(%)**	**21 (7%)**
Infected prosthetic grafts	12
Mycotic aneurysm/septic thrombophlebitis	5
Central line-associated bloodstream infection	4

**Table 2:** Baseline predisposition characteristics of overall cohort and five major site subgroups. *BMI* body mass index, *ESRD* end-stage renal disease

	Overall, ***n*** = 316	Abdominal, ***n*** = 140 (44%)	Pulmonary, ***n*** = 59 (19%)	S/ST, ***n*** = 56 (18%)	GU, ***n*** = 40 (12%)	Vascular, ***n*** = 21 (7%)	***p*** value
Male, *n* (%)	173 (55)	68 (49)	42 (71)	28 (50)	20 (50)	15 (71)	0.02
Age in years, median (25th, 75th)	62 (50,70)	63 (50,71)	62 (54,68)	56 (44,64)	61 (51,73)	69 (58,73)	0.03
Age ≥ 65 years, *n* (%)	125 (40)	60 (43)	22 (37)	12 (21)	18 (45)	13 (62)	< 0.01
Race, *n* (%)							0.39
Caucasian (White)	283 (90)	127 (91)	56 (95)	47 (84)	34 (85)	19 (90)	
African American	28 (9)	10 (7)	3 (5)	8 (14)	6 (15)	1 (5)	
Other	5 (1)	3 (2)	0 (0)	1 (2)	0 (0)	1 (5)	
Hispanic ethnicity	8 (3)	3 (2)	1 (2)	2 (4)	1 (3)	1 (4)	
BMI, median (25th, 75th)	29 (25,37)	28 (24,34)	29 (24,33)	35 (28,43)	34 (26,39)	28 (25,33)	< 0.01
Number of comorbidities							
1	65 (21)	28 (20)	14 (24)	14 (25)	7 (18)	2 (10)	0.61
2	65 (21)	34 (24)	12 (20)	9 (16)	7 (18)	3 (14)	0.69
≥ 3	134 (42)	57 (41)	23 (39)	23 (41)	17 (43)	14 (67)	0.25
Charlson comorbidity index, median (25th, 75th)	3 (1,5)	3 (1,5)	3 (1,4)	2 (1,4)	3 (1,5)	4 (3,7)	0.07
Type of comorbidities							
Hypertension	195 (62)	86 (61)	41 (70)	28 (50)	24 (60)	16 (77)	0.16
Diabetes	109 (34)	44 (31)	14 (24)	30 (54)	14 (35)	7 (33)	0.02
Coronary artery disease	80 (25)	32 (23)	16 (27)	11 (20)	10 (25)	11 (52)	0.07
Chronic lung disease	62 (20)	32 (23)	17 (29)	6 (11)	1 (2)	6 (29)	< 0.01
Morbid obesity	56 (18)	22 (16)	7 (12)	17 (30)	8 (20)	2 (10)	0.08
Chronic renal disease	44 (14)	17 (12)	7 (12)	9 (16)	6 (15)	5 (24)	0.60
ESRD	8 (3)	3 (2)	2 (3)	1 (2)	0 (0)	2 (10)	0.25
Atrial fibrillation	38 (12)	17 (12)	4 (7)	8 (14)	5 (12)	4 (19)	0.54
Peripheral artery disease	38 (12)	12 (9)	6 (10)	5 (9)	4 (10)	11 (53)	< 0.01
Heart failure	37 (12)	13 (9)	8 (14)	8 (14)	3 (8)	5 (24)	0.28
Prior Stroke	24 (8)	15 (11)	5 (8)	2 (4)	2 (5)	0 (0)	0.33
Substance abuse	21 (7)	11 (8)	5 (8)	3 (5)	0 (0)	2 (10)	0.32
Dementia	7 (2)	3 (2)	2 (3)	0 (0)	1 (2)	1 (5)	0.49
Liver cirrhosis	6 (2)	2 (1)	2 (3)	1 (2)	1 (2)	0 (0)	0.85
History of cancer, *n* (%)	82 (26)	46 (33)	14 (24)	9 (16)	10 (25)	3 (14)	0.10
Active cancer, *n* (%)	47 (15)	30 (21)	9 (15)	3 (5)	5 (12)	0 (0)	< 0.01
Reason for hospital admission, *n* (%)							
Active infection	188 (60)	93 (66)	4 (7)	51 (91)	23 (58)	17 (81)	< 0.01
Elective surgery	64 (20)	29 (21)	18 (31)	3 (5)	12 (30)	2 (9)	< 0.01
Trauma	33 (10)	5 (4)	25 (42)	1 (2)	1 (2)	1 (5)	< 0.01
Chronic health condition	31 (10)	13 (9)	12 (20)	1 (2)	4 (10)	1 (5)	0.02
Inter-facility hospital transfer, *n* (%)	129 (40.8)	64 (45.7)	19 (32.2)	21 (37.5)	6 (15)	19 (90.5)	< 0.01

Table 3 compares the characteristics of the inciting septic insult. For the overall cohort, baseline APACHE II and SOFA scores were high, consistent with need for ICU care. Over a quarter presented in septic shock and over two thirds required source control procedures. Roughly 40% had negative culture results. Positive cultures were predominantly gram-negative and polymicrobial. Two thirds of abdominal infections had sepsis on admission, and they had the highest lactate levels and higher APACHE II scores. Almost all required source control but less than half had positive cultures. Pulmonary infections were more likely to be hospital-acquired, had higher APACHE II scores, and infrequently required source control. S/ST and GU infections were more likely to be present on admission with lower APACHE II and 24-h SOFA scores. Most S/ST infections were present on admission and required emergency surgery within 24 h with a higher rate of positive cultures (half were polymicrobial). GU infections had high lactate levels (i.e., shock severity), but lower baseline APACHE II and SOFA scores (i.e., organ dysfunction). Less than half underwent source control procedures (almost all were non-invasive) and had a high rate of positive culture (70% were gram-negative). On the other hand, three fourths of vascular infections required source control (all invasive). They had the highest baseline APACHE II and SOFA scores and the highest rate of positive culture results.

**Table 3:** Characteristics of the inciting septic insult by site of infection. *APACHE* Acute Physiology Age Chronic Health Evaluation, *SOFA* Sequential Organ Failure Assessment

	Overall, ***n*** = 316	Abdominal, ***n*** = 140 (44%)	Pulmonary, ***n*** = 59 (19%)	S/ST, ***n*** = 56 (18%)	GU, ***n*** = 40 (12%)	Vascular, ***n*** = 21 (7%)	***p*** value
Sepsis present on admission (≤ 48 h), *n* (%)	204 (65)	94 (67)	17 (29)	49 (87)	31 (78)	13 (62)	< 0.01
Hospital-acquired sepsis (> 48 h), *n* (%)	112 (35)	46 (33)	42 (71)	7 (13)	9 (22)	8 (38)	< 0.01
Sepsis severity, *n* (%)							
Sepsis	95 (30)	38 (27)	18 (30)	14 (25)	16 (40)	9 (43)	0.31
Severe sepsis	138 (44)	61 (44)	28 (48)	25 (45)	16 (40)	8 (38)	0.94
Septic shock	83 (26)	41 (29)	13 (22)	17 (30)	8 (20)	4 (19)	0.59
Max lactate (within 24 h), median (25th, 75th)	2.1 (1.5,3.4)	2.7 (1.8,4.2)	1.5 (1.2,2.2)	2 (1.4,2.8)	2.5 (1.7,3.8)	2.1 (1.3,3)	< 0.01
Max lactate (within 24 h) > 2, *n* (%)	169 (54)	89 (64)	19 (32)	26 (46)	24 (60)	11 (52)	< 0.01
APACHE II score (24 h), median (25th, 75th)	17 (11,23)	17 (12, 23)	20 (14,25)	16 (10, 22)	13 (10,20)	21 (13,24)	0.02
SOFA score (24 h), median (25th, 75th)	7 (5,10)	7 (4, 10)	8 (6, 10)	6 (5, 10)	6 (4, 8)	8 (6,9)	< 0.05
Emergency surgery within 24 h, *n* (%)	160 (51)	64 (46)	27 (46)	45 (80)	15 (38)	9 (43)	< 0.01
Sepsis source control procedure, *n* (%)	215 (68)	124 (89)	4 (7)	53 (95)	18 (45)	16 (76)	< 0.01
Invasive procedures	152 (71)	87 (70)	3 (75)	45 (85)	1 (6)	16 (100)	< 0.01
Non-invasive procedures	63 (29)	37 (30)	1 (25)	8 (15)	17 (94)	0 (0)	< 0.01
Culture positive, *n* (%)	197 (62)	65 (46)	39 (66)	41 (73)	33 (83)	19 (90)	< 0.01
Bacterial—gram positive	47 (24)	14 (21)	10 (26)	14 (34)	2 (6)	7 (37)	< 0.01
Bacterial—gram negative	80 (41)	20 (31)	25 (64)	4 (10)	23 (70)	8 (42)	< 0.01
Fungal	10 (5)	5 (8)	0 (0)	1 (2)	3 (9)	1 (5)	0.20
Polymicrobial	60 (30)	26 (40)	4 (10)	22 (54)	5 (15)	3 (16)	< 0.01

Table 4 depicts ICU-related outcome and mortality. Overall, patients were in the ICU a median of 7 days. Two thirds required mechanical ventilation, 60% developed AKI, 15% developed MOF, and 10% died within 30 days. Over one third progressed into CCI, nearly half had a “poor” discharge disposition, and 21% died by 1 year. Abdominal infection patients had the highest rate of secondary infections. Most (86%) of the pulmonary infection patients required mechanical ventilation. This group had the longest ICU stays with the fewest ventilator-free days, and over half progressed into CCI. S/ST and GU infection patients had the lowest lengths of ICU stay, with high rates of rapid recovery and “good” discharge dispositions as well as notably low 30-day, 180-day, and 1-year mortality. On the other hand, vascular infection patients had the highest rate of “poor” discharge dispositions and the highest mortalities.

**Table 4:** ICU-related outcomes and mortality. *ICU* intensive care unit, *KDIGO* Kidney Disease Improving Global Outcomes

	Overall, ***n*** = 316	Abdominal, ***n*** = 140 (44%)	Pulmonary, ***n*** = 59 (19%)	S/ST, ***n*** = 56 (18%)	GU, ***n*** = 40 (12%)	Vascular, ***n*** = 21 (7%)	***p*** value
ICU days, median (25th, 75th)	7 (3, 17)	9 (3,18)	15 (8,21)	5 (3, 10)	3 (2,4)	9 (5,18)	< 0.01
Need for mechanical ventilation, *n* (%)	209 (66)	100 (71)	51 (86)	34 (61)	11 (28)	13 (62)	< 0.01
Ventilator-free days, median (25th, 75th)	27 (22,30)	27 (21,30)	25 (18,28)	28 (26,30)	30 (28,30)	28 (23,30)	< 0.01
Acute kidney injury, *n* (%)	188 (60)	79 (56)	31 (52)	39 (70)	25 (63)	14 (68)	0.32
KDIGO stage 1	80 (43)	23 (29)	19 (62)	18 (32)	15 (38)	5 (24)	0.01
KDIGO stage 2	65 (34)	33 (42)	6 (19)	16 (29)	7 (18)	3 (14)	0.10
KDIGO stage 3	43 (23)	23 (29)	6 (19)	5 (9)	3 (8)	6 (29)	0.12
Multiple organ failure, *n* (%)	48 (15)	24 (17)	11 (19)	5 (9)	4 (10)	4 (19)	0.43
Secondary infections/patient, mean (SD)	0.5 (0.9)	0.8 (1)	0.5 (0.8)	0.3 (0.6)	0.1 (0.4)	0.4 (0.7)	< 0.01
Secondary infections/100 hospital days	2.3 (4.8)	3.4 (6.2)	2.2 (4.0)	1.0 (2.2)	0.3 (1.0)	1.4 (2.3)	< 0.01
Clinical trajectory							
Early death (< 14 days), *n* (%)	14 (4)	9 (6)	1 (2)	1 (2)	1 (2)	2 (10)	0.32
Rapid recovery, *n* (%)	194 (62)	80 (57)	26 (44)	42 (75)	35 (88)	11 (52)	< 0.01
Chronic critical illness, *n* (%)	108 (34)	51 (37)	32 (54)	13 (23)	4 (10)	8 (38)	< 0.01
Discharge disposition, *n* (%)							
“Good” disposition	176 (56)	72 (51)	31 (53)	34 (61)	32 (80)	7 (33)	< 0.01
Home	60 (34)	23 (32)	6 (19)	8 (23)	22 (69)	1 (14)	
Homecare	97 (55)	43 (60)	17 (55)	23 (68)	9 (28)	5 (72)	
Rehab	19 (11)	6 (8)	8 (26)	3 (9)	1 (3)	1 (14)	
“Poor” disposition	140 (44)	68 (49)	28 (47)	22 (39)	8 (20)	14 (67)	< 0.01
Long-term care hospital	48 (34)	26 (38)	10 (36)	8 (36)	3 (38)	1 (7)	
Skilled nursing	48 (34)	21 (31)	8 (28)	9 (41)	3 (38)	7 (50)	
Another hospital	10 (7)	2 (3)	3 (11)	4 (18)	1 (12)	0 (0)	
Hospice	7 (5)	5 (7)	0 (0)	0 (0)	0 (0)	2 (14)	
Death	27 (20)	14 (21)	7 (25)	1 (5)	1 (12)	4 (29)	
30-day mortality, *n* (%)	31 (10)	19 (14)	5 (8)	1 (2)	2 (5)	4 (19)	0.03
180-day mortality, *n* (%)	59 (19)	31 (22)	11 (19)	4 (7)	5 (12)	8 (38)	0.01
1-year mortality, *n* (%)	67 (21)	36 (26)	12 (20)	5 (9)	5 (12)	9 (43)	< 0.01

**Fig. 1:**
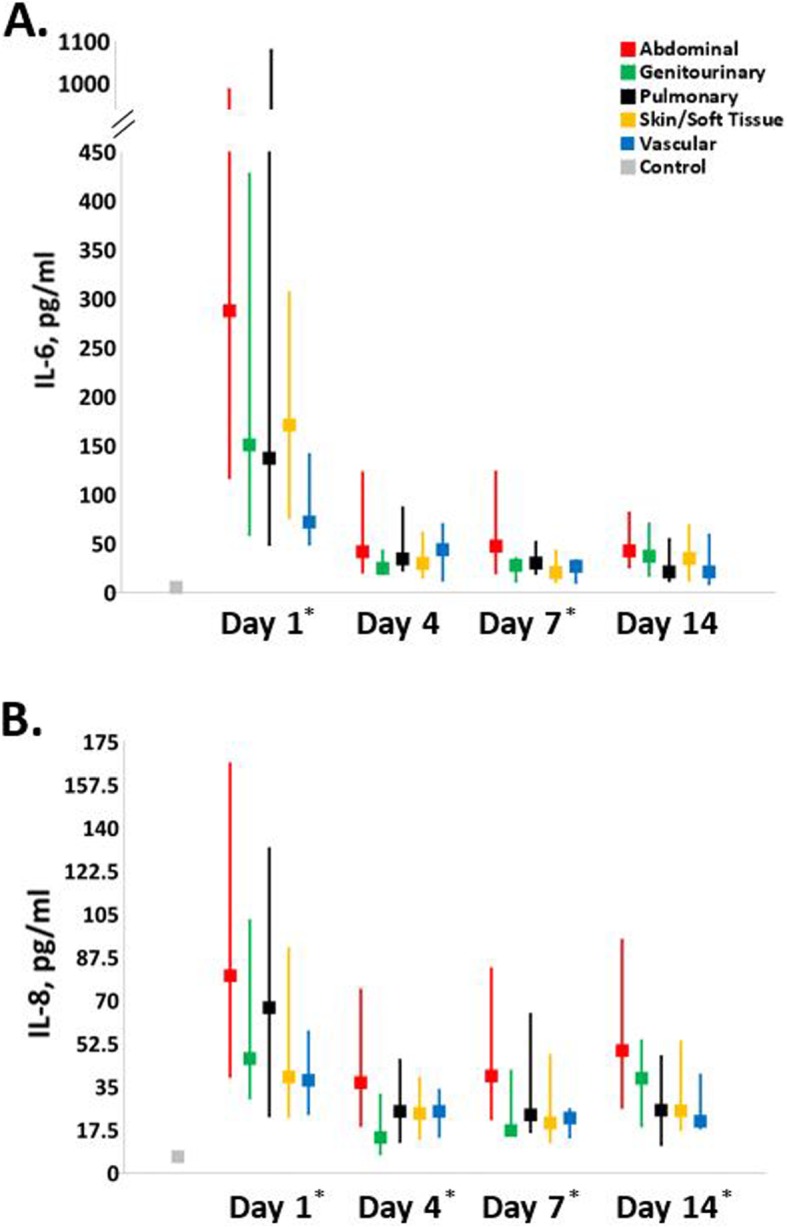
Serial biomarkers of proinflammation. Biomarkers of proinflammation (**a** interleukin-6, **b** interleukin-8) in each infection subgroup were measured for inpatients at 1, 4, 7, and 14 days post sepsis onset and compared to each other as well as matched healthy controls. An asterisk indicates significant intergroup differences (excluding controls, *p* < 0.05) at that time point

Figure 1 depicts the serial biomarker of proinflammation for the five subgroups. Compared to healthy control subjects, all subgroups have higher IL-6 (Fig. 1a) and IL-8 (Fig. 1b) levels at 1, 4, 7, and 14 days after sepsis onset (*p* <  0.01). Additionally, there were significant differences between subgroups in both IL-6 and IL-8 levels at the majority of time points. In all subgroups, IL-6 levels drop significantly from day 1 levels by either day 4 or 7 (*p* <  0.05), but still remain significantly elevated above control levels out to 14 days (*p* < 0.01). Comparatively, abdominal infections had a more persistent proinflammatory response.

**Fig. 2:**
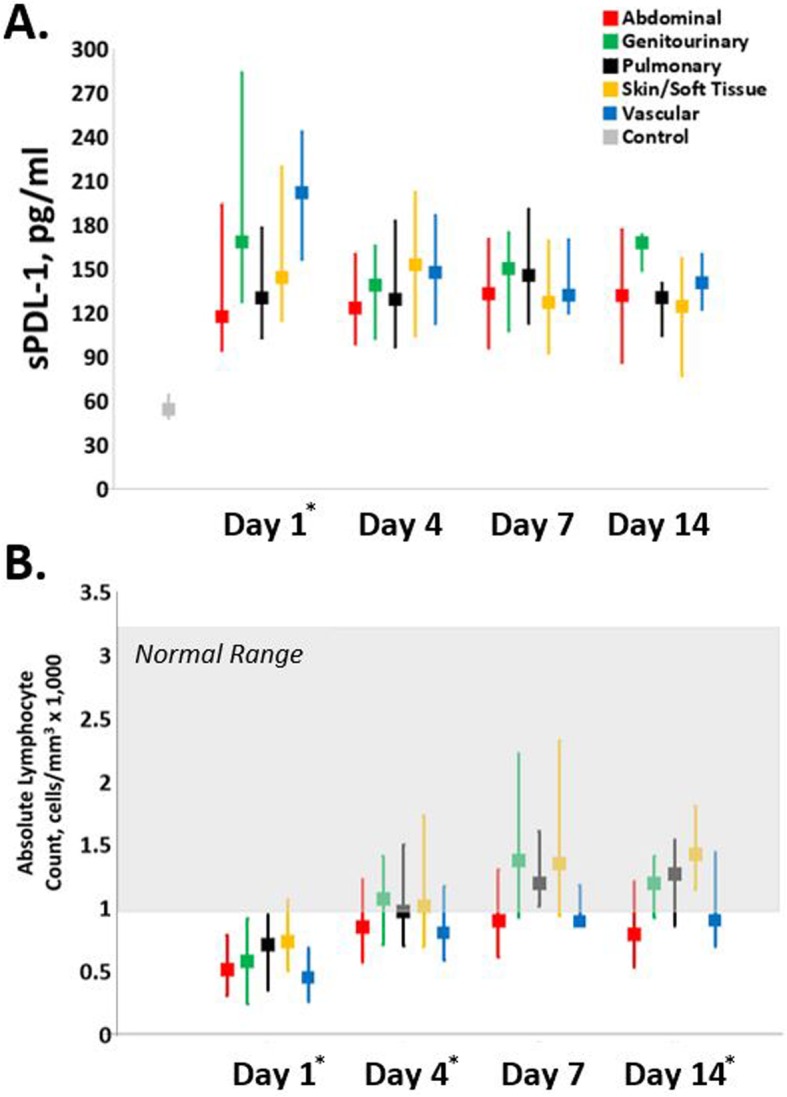
Serial biomarkers of immunosuppression. Biomarkers of immunosuppression (**a** soluble programmed death ligand-1, **b** absolute lymphocyte count) in each infection subgroup were measured for inpatients at 1, 4, 7, and 14 days post sepsis onset and compared to each other as well as matched healthy controls or normal reference range. An asterisk indicates significant intergroup differences (excluding controls, *p* < 0.05) at that time point

Figure 2 depicts biomarkers of immunosuppression by infection site subgroup. Compared to healthy control subjects, all subgroups had higher sPDL-1 concentrations (Fig. 2a) across all time points (*p* < 0.01). At day 1, there were significant intergroup differences between sPDL-1 levels, with vascular patients having significantly higher levels (*p* < 0.01) than abdominal and pulmonary subgroups. These values became statistically similar across all subgroups by day 4. On day 1, all subgroups have ALCs below the standard laboratory reference range (Fig. 2b). ALCs increase in all of the subgroups at the later time points, but remain below the normal range in the abdominal and vascular subgroups.

**Fig. 3:**
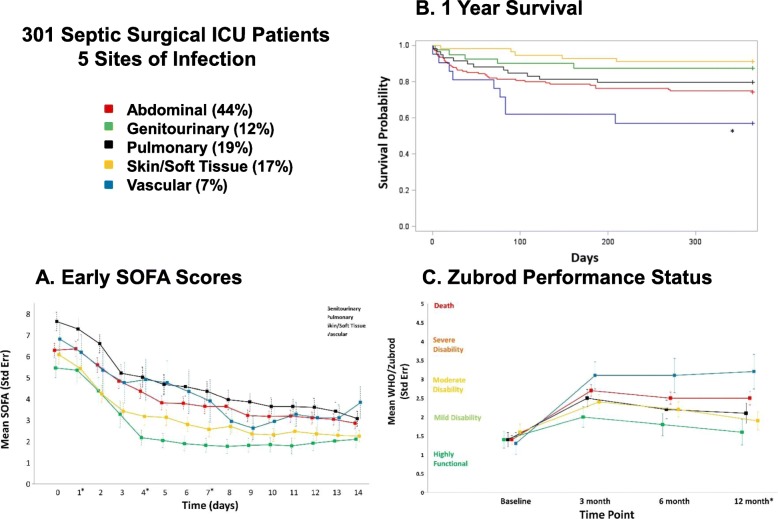
Summarizes the subgroups, their serial first 14-day SOFA scores, 1-year survival, and 1-year Zubrod performance status. **a** Depicts daily SOFA scores following sepsis onset (day 0) by subgroup. An asterisk indicates significant intergroup differences (*p* < 0.05) at that time point (only tested days 1, 4, 7, and 14). **b** Depicts 1-year survival by subgroup. An asterisk indicates significant intergroup differences (*p* < 0.005) by log-rank test. **c** Depicts performance status changes from baseline as measured by World Health Organization (WHO)/Zubrod score. Higher score indicates worse performance status (see the “Methods” section). An asterisk indicates significant intergroup differences at that time point

**Fig. 4:**
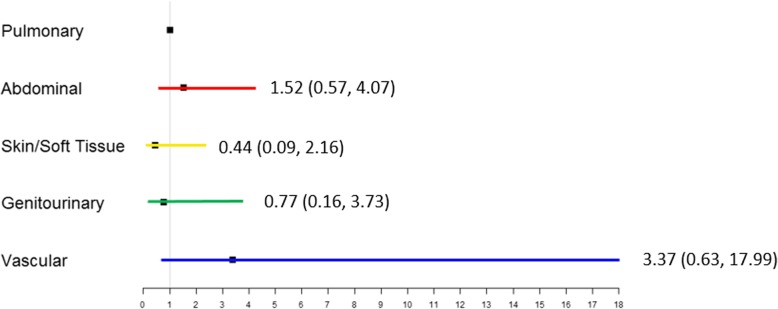
Depicts relative risk of 12-month mortality by sites of infection. Odds ratios and 95% confidence intervals are from multivariate logistic regression analyses done to assess the relative risk of 12-month mortality for abdominal, S/ST, GU, and vascular infections compared to pulmonary infections

Figure 3 summarizes the subgroups, their serial first 14-day SOFA scores, 1-year survival, and 1-year Zubrod performance status. On day 1, all of the groups have high SOFA scores with the highest in pulmonary and lowest in GU patients (Fig. 3a). SOFA deceased in all groups by day 4 but were notably lower in patients with S/ST and GU infections. There were significant intergroup differences in SOFA score at the tested time points of 1, 4, and 7 days. Figure 3b depicts 1-year survival curves by infection site subgroup. While S/ST and GU had the highest 1-year survival (91% and 87%, respectively), the vascular subgroup had the worst at 57%. Long-term performance status was measured by WHO/Zubrod scores (Fig. 3c). At baseline, there were no significant intergroup differences in performance status, with patients on average being ambulatory and able to carry out activities of daily living. Compared to their respective baselines, all groups had significantly higher Zubrod scores at 3 months (*p* < 0.05). However, while the S/ST and GU groups showed improvement toward their baseline (by 6 and 12 months, respectively), vascular, abdominal, and pulmonary groups remained significantly elevated through 12 months. Figure 4 depicts the results of multivariate logistic regression analyses to assess relative risk of 12-month mortality by sites of infection. In comparison to pulmonary infections, the 95% confidence intervals for abdominal, S/ST, GU, and vascular infections are wide and there are no statistically significant differences in the odds ratios.

## Discussion

The major finding of this study is that notable phenotypic heterogeneity exists in surgical sepsis patients when categorized by anatomic site of infection. This study is also unique in its inclusion of comparisons of the host response (serial biomarkers of immunity and organ dysfunction) as well as long-term outcomes (functional status and 1-year mortality) of the subgroups. The largest subgroup was abdominal sepsis; most of these patients had sepsis present on admission and required source control. These patients experienced robust proinflammation that persisted with prolonged immunosuppression (with more frequent secondary infections). Their initial high SOFA score persisted in the ICU and one third progressed into the CCI trajectory. At 12 months, their impaired functional status had not improved and they had the second-worst survival. On the other hand, pulmonary infections (the next most frequent) were principally hospital-acquired pneumonia that occurred in trauma and elective surgery patients requiring mechanical ventilation who had a high incidence of chronic lung disease. They experienced a similar robust proinflammatory response but immunosuppression normalized faster (with less secondary infections). Over half progressed into CCI, less than half were discharged to home and long-term outcomes were similarly dismal outcomes. However, in these patients, it is unclear whether the pulmonary infections were the cause of new organ dysfunction or just a symptom of immunosuppression in patients with organ dysfunction induced by their inciting traumatic or surgical insults [32]. S/ST infections (predominantly necrotizing) occurred in younger obese individuals who had high rate of diabetes. Their proinflammation persisted, but organ dysfunction normalized faster. Three-quarters of these patients rapidly recovered with a low 30-day mortality and their long-term functional status and survival were notably better. GU infections (predominantly complicated UTIs) had initial robust proinflammation (that resolved quickly) but lower SOFA scores (that resolved quickly). Of note, 88% rapidly recovered and they had the best 12-month survival and functional status. Vascular infection patients were older males, had the highest comorbidity burden, and were more likely to be transferred from other hospitals. Proinflammation was notably blunted compared to other infections and baseline immunosuppression persisted. They had prolonged organ dysfunction and the worst 12-month survival with notably poor functional outcomes.

Previous studies looking at the role site of infection in outcomes after sepsis principally focused on hospital mortality [12–15, 33]. From these reports, it can be concluded that site of infection does have an independent effect on hospital mortality. Despite the low 30-day mortalities observed in this study (ranging 14% in abdominal to 2% in S/ST), the relative differences by site are consistent with hospital mortalities from the previous published reports. A systematic review of studies from 2001 to 2014 found that pulmonary infections had the worst in hospital mortality [13]. When pulmonary infections were used as the reference, GU and S/ST infections had substantially lower mortality. In the majority of the studies, abdominal infections were associated with lower mortality than pneumonia, but in some studies, mortality was notably higher. This is likely due to the differences in the frequency and relative mortality for the types of abdominal infections included in the specific studies. In a recent study that included 8000 septic adults, patients were categorized into 20 primary sources of infection, and pulmonary infections were used as the reference group [12]. After adjusting for differences in baseline characteristics and downstream responses, it was found that ischemic bowel and the category of “other intra-abdominal infections” had the highest mortality (with odds ratios of 2 to 3), while specific types of abdominal infections (including enterocolitis/diverticulitis, cholangitis/cholecystitis, and peritonitis/abscess/small bowel obstruction) had lower mortality (with odds ratios less than 1). The more recent study by Jeganathan et al. found that pulmonary infections had the highest mortality (28%), with abdominal infection having intermediate mortality (15%) and GU the lowest (7%) [14]. In addition to hospital mortality, this report compared ICU outcomes by site of infection. Similar to our patients, pulmonary had the highest rate of organ failure and GU and S/ST had the lowest. However, most pulmonary infections in this report were community-acquired and thus difficult to compare to our cohort with principally hospital-acquired pneumonia.

Multiple factors likely contributed to the subgroup differences observed in this study. First, baseline predisposition places patients at increased risk for specific sites of infection. This was significant in our S/ST (with diabetes), pulmonary (with chronic lung disease), and vascular (with advanced age with cardiovascular disease) sepsis patients. Sepsis has also been called the “quintessential disease of the elderly.” In a separate report from this cohort focused on long-term functional and cognitive testing [34]. It demonstrated when compared to the young (≤ 45 years) and middle-aged (46–64 years) patients, older patients (defined as ≥ 65 years) had significantly worse long-term outcomes and were less likely to recover. Interestingly, in logistic regression analyses, it was found that age ≥ 65 years was not an independent predictor. This was true when we further dichotomized this older cohort into subgroups of patients who were 65–74 versus ≥ 75 years old. We believe this is true because chronologic age is not a good reflection of physiologic age. In this manuscript, we discuss the multiple baseline factors and the contribution of alterations in immune response with aging. Second, the difficulty in diagnosis can affect severity at presentation and delay in needed interventions. This is most relevant with abdominal infections, especially in the elderly where as a result of blunted SIRS and confounding comorbid disease, their presentations are atypical. A third issue is a delay in interventions related to transfer from outside hospitals where the diagnosis of sepsis may not be considered prior to transfer or early sepsis care bundles may not have been effectively implemented. This is particularly important in surgical patients where transfer can significantly delay source control interventions (e.g., NSTI debridement) which are known to have substantial impact on outcomes [35]. Moreover, protective barriers and defense mechanisms are unique to each anatomic as is the burden of organisms and type of bacterial infection. We did identify significant differences in the types of organisms in culture-positive patients, but given the small numbers in the subgroups, it is difficult to ascertain the impact on clinical progression. Lastly, sepsis is a disease of dysregulated immunity and serial biomarkers in our subgroups reveal different responses. Some of this relates to baseline immune status. For example, the vascular patients were immunosuppressed and had a notably blunted proinflammatory response and the pulmonary infections were primarily ventilator pneumonia in patients who had a recent inflammatory insult. The relative ease of source control may also have played a role. This likely accounts the rapid recovery and better outcomes with GU infections (with high rates of non-invasive procedures) in contrast to abdominal infections where invasive laparotomies (sometimes multiple) were required to control ongoing peritonitis.

The observations made in this study should assist clinicians in decision-making and prognostication. As noted above, at presentation comorbidity status should prompt clinical suspicion for specific types of infection. Additionally, based on the site of infection, clinicians can better advise patients and their families on anticipated clinical trajectory, in particular the chances of progressing into CCI and its implications for long-term recovery. Moreover, failure to follow the anticipated trajectory should prompt suspicion that something is not right. The initial empiric antimicrobial coverage may need to be adjusted (increase dosing or change in agents) or an additional source control intervention may be required. Sepsis patients are also prone to develop secondary infections which are site-dependent and occur later in the clinical course.

This study also provides information relevant to future clinical trial design. The low-observed 30-day mortality supports the current consensus that future study endpoints need to focus to long-term outcomes [36]. Additionally, given that sepsis is believed to be due to a dysregulated immune response, our data indicate that these subgroups should be studied independently. They had notable differences in both in the pattern and duration of immune biomarkers that correlated with differences in the resolution of organ dysfunction and long-term outcomes. The NAGMSC Working Group on Sepsis recently convened by the US National Institute on Health emphasized that phenotypic heterogeneity is a major challenge in sepsis and that similar to cancer, the spectrum of sepsis needs to be characterized and treated based on the underlying biology of specific phenotypes [8]. A recent *JAMA* report by Seymour et al. supports this concept [10]. From our data, we believe abdominal infections would be the best subgroup to study. They represent a major challenge based on their high frequency and poor outcomes. They also best represent the clinical phenotype of what we call PICS-CCI [24, 25]. The challenge will be to identify early those abdominal sepsis patients (roughly one third) who have a likelihood to progress into CCI and thus potentially benefit from novel multimodality interventions directed at the underlying pathobiology of PICS.

### Limitations

First, this is an observational study, and thus, it is difficult to differentiate causation from correlation. Second, this study was performed at a single tertiary regional medical center that receives a high number of inter-facility hospital transfers. This confounds the generalizability of the observations. For example, the vascular infections were referred due to the unique local expertise of our vascular surgery group. This was a relatively small subgroup, but they were very different in their predisposition, immune responses, and long-term outcomes. Future studies need to be done at multiple institutions so that a larger population of vascular sepsis patients can be characterized. A third limitation is that we used the 2001 consensus guidelines diagnostic criteria (referred to as Sepsis-2) as entry criteria because that was the standard when we started enrollment in 2015 [37]. In 2016, the Sepsis-3 criteria were described and have gained popularity [38]. To address concerns that we were not using Sepsis-3 definitions, we performed interim analysis of our database in 2017 and found that 7% of the study patients that were classified as sepsis by Sepsis-2 criteria would be classified as only having an infection by Sepsis-3 criteria (because of the lack of attributable organ dysfunction) [39]. However, it is important to note that these were ICU patients who had to exceed the physiologic derangement threshold quantitated by the MEWS-SRS screening tool to be entered the study. When we compared various equivalent strata of Sepsis-2 and Sepsis-3 cohorts, we found no significant difference in immune biomarkers, SOFA scores, inpatient clinical outcomes, discharge disposition, mortality, and long-term Zubrod performance status. The fourth limitation is the consolidation of the various types of infections into five categories. This was based on the literature and was done to simplify the analysis, but likely generated bias (as discussed above with the abdominal infections). Fifth, comorbid disease plays an important role in the predisposition and outcomes of sepsis in aging patients. We obtained comorbidity data by concurrent chart review, but a more in-depth interview with the patient/family plus specific biomarkers (such as HbA1C for diabetes) would have allowed quantitation of poor control or severity. Sixth, 30% of the patients were admitted for trauma or elective surgery and subsequently developed sepsis. Their admission insult likely placed them at increased risk for infection, caused organ dysfunctions, and altered the immune responses. Comparing these hospital-acquired infection patients to those who were admitted with infections warrants further study. Seventh, biomarkers of immune responses and organ dysfunction were measured during hospitalization and documented persistent derangements in the CCI patients, but it would be interesting to obtain these after hospital discharge to document resolution. A recent study by Yende et al. showed that a subset of sepsis patients has a persistent elevation of proinflammation and immunosuppression up to a year, and this was independently associated with poor long-term outcomes [40].

## Conclusions

In this well-characterized cohort of surgical sepsis patients categorized by site of infection, there were notable differences found in baseline predisposition, immune responses, organ dysfunction resolution, ICU trajectories, and long-term clinical outcomes. Understanding of this phenotypic heterogeneity will assist in decision-making and prognostication. Additionally, further clarification of the underlying pathobiology of different subgroups will be needed to develop precise interventions and design future clinical trials.

## Data Availability

The datasets used and/or analyzed during the current study are available from the corresponding author on reasonable request.
